# Prediction models for major adverse cardiovascular events following ST-segment elevation myocardial infarction and subgroup-specific performance

**DOI:** 10.3389/fcvm.2023.1181424

**Published:** 2023-04-25

**Authors:** Weiyao Chen, Xin Tan, Xiaoyu Du, Qin Li, Meng Yuan, Hui Ni, Yuan Wang, Jie Du

**Affiliations:** ^1^ Department of Physiology and Pathophysiology, School of Basic Medical Sciences, Tianjin Medical University, Tianjin, China; ^2^ Beijing Institute of Heart Lung and Blood Vessel Diseases, Beijing Anzhen Hospital, Capital Medical University, Beijing, China; ^3^ The Key Laboratory of Remodeling-Related Cardiovascular Diseases, Ministry of Education, Beijing Collaborative Innovation Centre for Cardiovascular Disorders, Beijing, China; ^4^ Center for Cardiovascular Medicine, The First Hospital of Jilin University, Changchun, China

**Keywords:** ST-segment elevation myocardial infarction, machine learning, GRACE score, sex, hypertension, diabetes mellitus

## Abstract

**Background:**

ST-segment elevation myocardial infarction (STEMI) patients are at a high residual risk of major adverse cardiovascular events (MACEs) after revascularization. Risk factors modify prognostic risk in distinct ways in different STEMI subpopulations. We developed a MACEs prediction model in patients with STEMI and examined its performance across subgroups.

**Methods:**

Machine-learning models based on 63 clinical features were trained in patients with STEMI who underwent PCI. The best-performing model (the iPROMPT score) was further validated in an external cohort. Its predictive value and variable contribution were studied in the entire population and subgroups.

**Results:**

Over 2.56 and 2.84 years, 5.0% and 8.33% of patients experienced MACEs in the derivation and external validation cohorts, respectively. The iPROMPT score predictors were ST-segment deviation, brain natriuretic peptide (BNP), low-density lipoprotein cholesterol (LDL-C), estimated glomerular filtration rate (eGFR), age, hemoglobin, and white blood cell (WBC) count. The iPROMPT score improved the predictive value of the existing risk score, with an increase in the area under the curve to 0.837 [95% confidence interval (CI): 0.784–0.889] in the derivation cohort and 0.730 (95% CI: 0.293–1.162) in the external validation cohort. Comparable performance was observed between subgroups. The ST-segment deviation was the most important predictor, followed by LDL-C in hypertensive patients, BNP in males, WBC count in females with diabetes mellitus, and eGFR in patients without diabetes mellitus. Hemoglobin was the top predictor in non-hypertensive patients.

**Conclusion:**

The iPROMPT score predicts long-term MACEs following STEMI and provides insights into the pathophysiological mechanisms for subgroup differences.

## 1. Introduction

Despite the improvement in prognosis achieved by reopening occluded vessels *via* percutaneous coronary intervention (PCI), patients with ST-segment elevation myocardial infarction (STEMI) are at a high risk of subsequent major adverse cardiovascular events (MACEs) (1, 2). The Global Registry of Acute Coronary Events (GRACE) risk score, which includes age, systolic blood pressure, heart rate, serum creatinine concentration, Killip class, cardiac arrest, elevated cardiac biomarkers, and ST-segment deviation, is a guideline-recommended risk stratification tool for patients with STEMI that is used to determine short-term prognosis (3). However, vital in patients with STEMI is the long-term prognosis, with 7.9%–10.1% of patients experiencing MACEs within 1 year (2, 4). Between 6 months and 2 years after myocardial infarction (MI) onset, cardiac mortality accounts for half of the total mortality, whereas cardiovascular death due to reinfarction (median 8 months) is the most frequent cause of mortality (5). The prediction of long-term prognosis is critical for risk stratification and disease management in patients with STEMI who undergo PCI. After revascularization, patients with STEMI experience a series of pathophysiological changes, including inflammation, neuroendocrine deregulation, coagulation abnormalities, and metabolic alterations, leading to long-term MACEs (1). Therefore, long-term prediction models based on the pathophysiological processes that follow STEMI are needed.

STEMI represents a heterogeneous disease that originates from a complex interaction between genetic and environmental factors. The prognosis of STEMI varies among patients and is largely dependent on risk factors (6). Previous studies have demonstrated that multiple risk factors and their interactions are associated with the long-term prognosis of patients with STEMI treated with primary PCI (7, 8). Patients with STEMI who are stratified into subgroups according to these risk factors have distinct clinical profiles, resulting in different MACEs risks (9–11). Therefore, additional subgroup-specific evaluation to predict the long-term prognosis of patients following STEMI is required to achieve effective management in different patient subgroups.

Early assessment and risk stratification during the acute phase of STEMI are essential. Clinical and biochemical characteristics can be obtained rapidly after admission at both primary and tertiary hospitals. These indicators reflect inherent pathophysiological processes and can be instrumental in predicting the long-term risk of MACEs in patients with STEMI (12). Numerous correlated clinical variables are considered, which adds to the complexity of the assessment and the difficulty in making decisions about individual patients. To overcome these limitations, data-driven approaches using machine learning (ML) algorithms have been proposed. These algorithms can process and learn from large, complex, and multidimensional clinical data to develop effective predictive models (13).

In this study, we aimed to design an accurate prediction model that incorporates variables that are readily available in clinical practice to assess the individual risk of long-term MACEs in patients with STEMI who undergo PCI. Moreover, we aimed to assess the model performance and variable contribution in various subgroups stratified by different risk factors to broaden the understanding of the pathophysiological processes following STEMI, especially in specific subgroups.

## 2. Materials and methods

### 2.1. Study design and participants

The retrospective cohort with 1,007 STEMI patients who underwent PCI was designated as the derivation cohort to develop the ML models. The retrospective cohort was obtained from the Coronary artery disease database of Beijing Anzhen Hospital from August 2018 to August 2019 (Supplementary Figure S1). To assess the performance of the ML models, an external validation cohort of 240 patients with STEMI who attended the First Hospital of Ji Lin University from January 2014 to January 2017 was included. These patients were identified from the Registry Study of Genetics and Biomarkers of Acute Coronary Syndrome (ARSGB-ACS, NCT03752515). The study protocol conformed to the ethical guidelines of the 1975 Declaration of Helsinki as reflected in *a priori* approval by the human research committee of Beijing Anzhen Hospital. In the present study, we reported our findings following the guidelines of the Transparent Reporting of a Multivariable Prediction Model for Individual Prognosis or Diagnosis (TRIPOD) statement (14). Data were routinely collected from the electronic medical records database by a multicenter research platform.

All patients aged >18 years with a definite discharge diagnosis of STEMI who underwent PCI were included. The diagnostic criteria followed the Joint European Society of Cardiology/American College of Cardiology Foundation/American Heart Association/World Heart Federation Task Force for the Universal Definition of Myocardial Infarction (15). Patients with in-hospital death; abnormal left ventricular function [left ventricular ejection fraction (LVEF) of ≤30%]; severe kidney dysfunction [estimated glomerular filtration rate (eGFR) of ≤30 ml/min]; or missing data on LVEF, eGFR, or GRACE score were excluded. The study inclusion and exclusion criteria are summarized in Supplementary Figure S1.

### 2.2. Outcomes

The primary outcome was MACEs, including all-cause mortality, non-fatal MI, and non-fatal ischemic stroke. We obtained outcome data from patient visits, medical records, and telephone interviews. A total of 136 patients without available follow-up information due to lack of contact information or withdrawal were excluded.

### 2.3. Feature selection and data preprocessing

We selected reliable and easily collectable candidate risk factors that were present before or during the index hospitalization based on literature reviews. Sixty-three candidate variables were collected, including clinical characteristics, echocardiographic parameters, and laboratory indices. As the availability of brain natriuretic peptide (BNP) and N-terminal proBNP (NT-proBNP) concentration analyses differ between institutions, BNP and NT-proBNP concentrations were log-transformed and converted using a previously cited formula (16), as follows: log BNP = 0.28 + 0.66 × log NT-proBNP. The GRACE risk score was used (3, 17). Data quality control was performed before data analysis, and missing values were imputed using multiple imputations with 10 imputations (missing values are shown in Supplementary Tables S1, S2). The final imputed value was an average of 10 imputations.

### 2.4. Model development and validation

**Figure 1:**
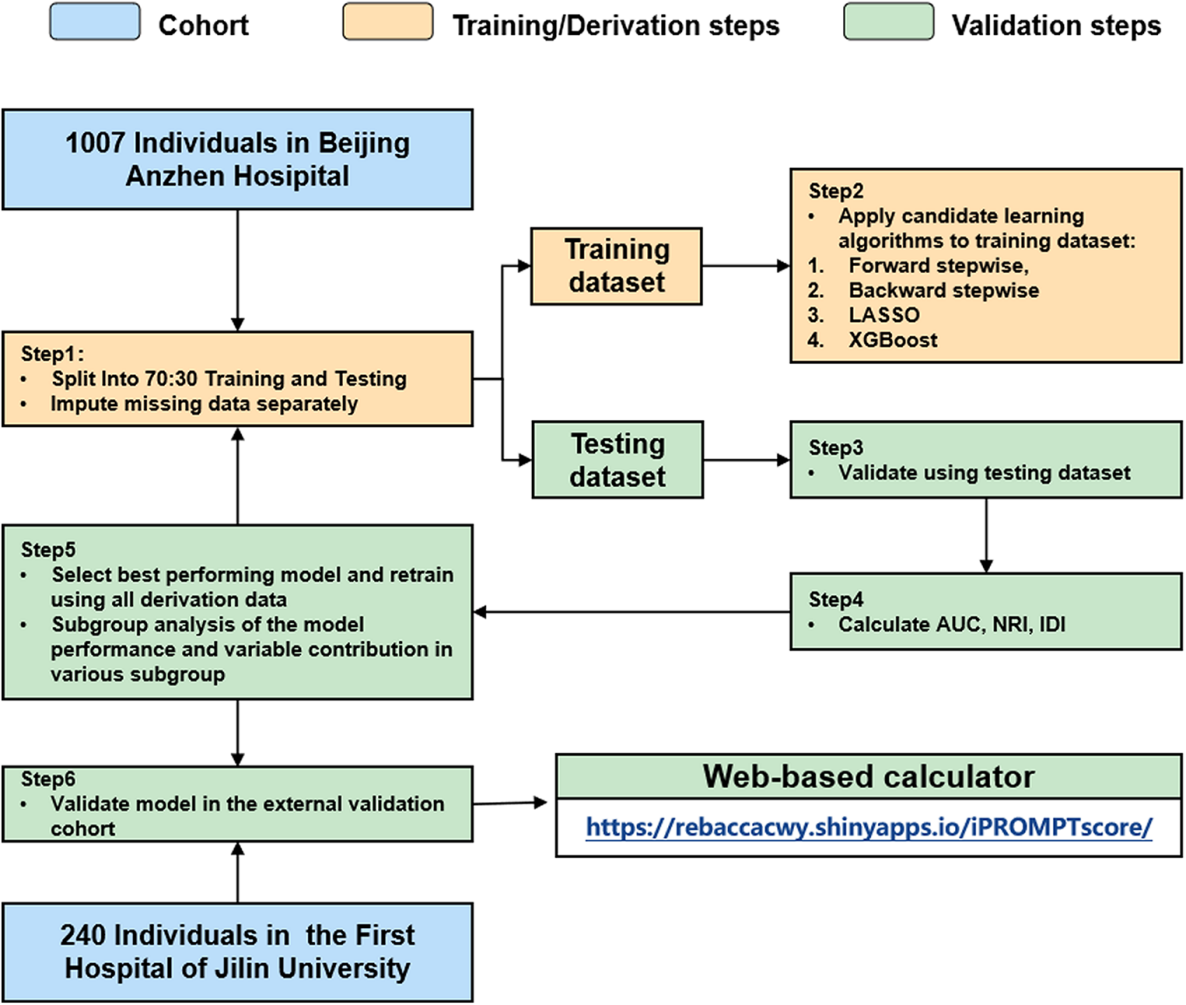
Analysis overview for identifying best-performing risk prediction model. AUC, area under the curve; NRI, net reclassification index; IDI, integrated discrimination index.

The derivation cohort was randomly split into the training [*n* = 705 (70%)] and testing [*n* = 302 (30%)] datasets (Figure 1). The model was trained on each imputed training dataset. The forward stepwise, backward stepwise, Least Absolute Shrinkage and Selection Operator (LASSO) regression and XGBoost methods were used to screen the variables, and the logistic regression analysis was used for modeling. Continuous variables were modeled with restricted cubic splines to relax the assumption of the linear effect and flexibly allow for non-linear effects. Receiver operating characteristic (ROC) curves and the area under the ROC curve (AUC) were used to estimate model discrimination. We compared the diagnostic performance between the selected model and the GRACE score by calculating the AUC, net reclassification improvement (NRI), and integrated discriminatory index (IDI). The model calibration was further explored by comparing the predicted and observed probabilities across predicted risk quintiles. The decision curve analysis (DCA) was also performed to estimate the clinical usefulness and benefits. The model with the best performance and comprehensive evaluation in both the training and testing datasets was chosen for further external validation in the validation cohort.

### 2.5. Subgroup analysis and variable contribution in the model

Subgroup analyses were conducted based on prespecified covariates, including sex, hypertension, and diabetes mellitus. The importance of a selected variable describes how much the variable contributes to the improvement in the model, which was assessed by examining the decrease in the AUC of the fitted model when the test variable was replaced. A greater decrement in the AUC was considered to indicate a greater contribution of the replaced variable to the model performance.

### 2.6. Statistical analysis

Categorical variables are presented as percentages. Normally distributed continuous variables are presented as the mean (standard deviation), while non-normally distributed continuous variables are presented as the median (interquartile range). Continuous variables were compared using the bilateral independent *t*-test or the Wilcoxon rank-sum test, while categorical variables were compared using the chi-square test or Fisher's exact test. We analyzed the predictor variable effects using the odds ratio values and beta coefficients in the model. In addition, a web-based dynamic nomogram was developed (Supplementary Figure S4). Statistical analyses were performed using Stata 16.1 (Stata Corp., College Station, TX, US) and R v4.0.3 (R Foundation for Statistical Computing, Vienna, Austria; packages: pROC, PredictABEL, ggDCA, rms, DynNom, Shiny).

## 3. Results

### 3.1. Baseline characteristics

After applying the inclusion and exclusion criteria, 1,007 patients were included in the derivation cohort. During the median follow-up period of 2.56 years, 50 patients (5.0%) experienced MACEs, including 30 patients (3.0%) with all-cause death, 9 patients (0.9%) with non-fatal MI, and 11 patients (1.09%) with non-fatal stroke.

Patients were divided into two groups according to their clinical outcomes during follow-up. Compared with the non-MACEs group, the MACEs group had a greater burden of cardiovascular risk factors, including older age, and a higher prevalence of smoking, hypertension, and ischemic stroke. Moreover, these patients experienced a greater injury at onset. Descriptive statistics for the continuous and categorical variables are summarized in Table 1.

**Table 1:** Clinical characteristics between MACEs group and non-MACEs group.

	Non-MACEs (*N* = 957)	MACEs (*N* = 50)	*P* value
Demographic characteristic			
Male gender, *n* (%)	791 (82.7%)	33 (66.0%)	0.003
Age, years	57.31 ± 10.86	65.88 ± 11.25	<0.001
BMI, kg/m2	25.86 ± 3.16	25.14 ± 3.12	0.153
Admission status			
SBP, mmHg	121.43 ± 17.59	125.78 ± 19.48	0.128
DBP, mmHg	74.49 ± 11.46	74.64 ± 14.39	0.944
Heart rate, bpm	73.22 ± 11.62	77.02 ± 17.19	0.128
ST-segment deviation, *n* (%)	311 (32.5%)	33 (66.0%)	<0.001
Killip classification, *n* (%)			
1	833 (87.0%)	39 (78.0%)	0.33
2	93 (9.72%)	8 (16.0%)	
3	9 (0.94%)	1 (2.00%)	
4	22 (2.30%)	2 (4.00%)	
Cardiac arrest, *n* (%)	18 (1.88%)	2 (4.00%)	0.295
Multivessel disease, *n* (%)	714 (74.6%)	36 (72.0%)	0.806
Personal history			
Hypertension, *n* (%)	543 (56.7%)	39 (78.0%)	0.003
Diabetes mellitus, *n* (%)	300 (31.3%)	21 (42.0%)	0.115
Hyperlipidemia, *n* (%)	840 (87.8%)	46 (92.0%)	0.37
Prior myocardial infarction, *n* (%)	61 (6.37%)	3 (6.00%)	1
Prior CVD, *n* (%)	109 (11.4%)	8 (16.0%)	0.321
Prior ischemic stroke, *n* (%)	64 (6.69%)	9 (18.0%)	0.007
Atrial fibrillation, *n* (%)	27 (2.82%)	3 (6.00%)	0.183
Prior PCI, *n* (%)	70 (7.31%)	5 (10.0%)	0.412
Prior CABG, *n* (%)	6 (0.63%)	0 (0.00%)	1
Smoking, *n* (%)	510 (53.3%)	17 (34.0%)	0.008
Laboratory tests			
cTnI, ng/L	0.78 (0.05–11.6)	2.63 (0.10–12.0)	0.418
CK-MB, ng/ml	56.17 ± 93.23	48.74 ± 73.73	0.497
BNP, pg/ml	213.02 ± 240.81	396.29 ± 349.84	0.02
Urea, mg/dl	6.03 ± 3.45	6.83 ± 3.35	0.109
eGFR (CKD-EPI)	95.57 ± 15.14	82.95 ± 19.83	<0.001
Creatinine, μmol/L	76.5 (65.8–87.4)	79.5 (67.8–103)	0.075
ALT, U/L	39.99 ± 36.93	29.96 ± 24.20	0.007
AST, U/L	61.90 ± 75.42	54.68 ± 61.42	0.427
Plasma sodium, mmol/L	139.20 ± 5.05	138.68 ± 3.11	0.269
Plasma potassium, mmol/L	4.12 ± 0.45	4.21 ± 0.45	0.186
Plasma chloride, mmol/L	100.59 ± 3.82	101.12 ± 4.37	0.404
HbA1c, %	6.58 ± 1.56	6.79 ± 1.58	0.406
Fasting glucose, mmol/L	8.53 ± 5.94	9.32 ± 3.92	0.185
Total cholesterol, mmol/L	4.36 ± 1.12	4.08 ± 1.21	0.125
Triglyceride, mmol/L	1.82 ± 1.46	1.77 ± 2.14	0.86
HDL-C, mmol/L	1.01 ± 0.25	1.00 ± 0.29	0.659
LDL-C, mmol/L	2.71 ± 0.94	2.41 ± 0.74	0.009
Homocysteine, mmol/L	15.96 ± 9.04	17.14 ± 8.18	0.369
Uric acid, μmol/L	360.42 ± 97.47	355.74 ± 114.90	0.779
White blood cell count, n/dl	9.03 ± 2.94	8.94 ± 3.59	0.864
Red blood cell count, n/dl	4.85 ± 4.15	4.47 ± 0.61	0.017
Platelet, n/dl	238.19 ± 66.19	240.76 ± 59.49	0.768
Hemoglobin, g/L	146.91 ± 15.47	137.08 ± 18.76	0.001
Neutrophil count, n/dl	6.65 ± 2.87	6.58 ± 3.35	0.883
Lymphocyte count, n/dl	1.79 ± 0.73	1.75 ± 0.79	0.719
hsCRP, mg/L	4.60 (1.63–15.2)	11.7 (2.46–25.0)	0.021
Imaging variables			
Ejection fraction, %	55.22 ± 8.46	53.74 ± 10.11	0.315
LVEDi, mm	48.82 ± 4.95	48.60 ± 5.92	0.799

For continuous variables, non-normal variables were expressed as the median [interquartile range (IQR)], and normal variables were expressed as the mean [standard deviation (SD)]. Categorical variables are expressed in *N* (%). *P* ≤ 0.05 was considered statistically significant. BMI, body mass index; SBP, systolic blood pressure; DBP, diastolic blood pressure; PCI, percutanous coronary intervention; CABG, coronary artery bypass grafting; cTnI, cardiac troponin I; CK-MB, creatine kinase MB; BNP, B-type natriuretic peptide; eGFR, estimate glomerular filtration rate; ALT, alanine aminotransferase; AST, aspartate aminotransferase; HDL-C, high-density lipoprotein cholesterol; LDL-C, low-density lipoprotein cholesterol; HbA1c, glycated hemoglobin A1c; hsCRP, high sensitive C reaction protein; LVEDi, left ventricle end-diastolic volume index.

### 3.2. Predictor variables and ML model construction

The risk prediction models were developed using XGBoost, forward stepwise, backward stepwise, and LASSO regression in the training dataset. Overall, all models yielded good discrimination (AUC: 80.3%–85.1% and 74.7%–81.9% in the training and testing datasets, respectively). The model performance is displayed in Supplementary Figure S1 and Supplementary Table S3. The best-performing model (the iPROMPT score) was established based on the features selected by the LASSO algorithm.

### 3.3. Performance of the iPROMPT score in the derivation cohort

**Figure 2:**
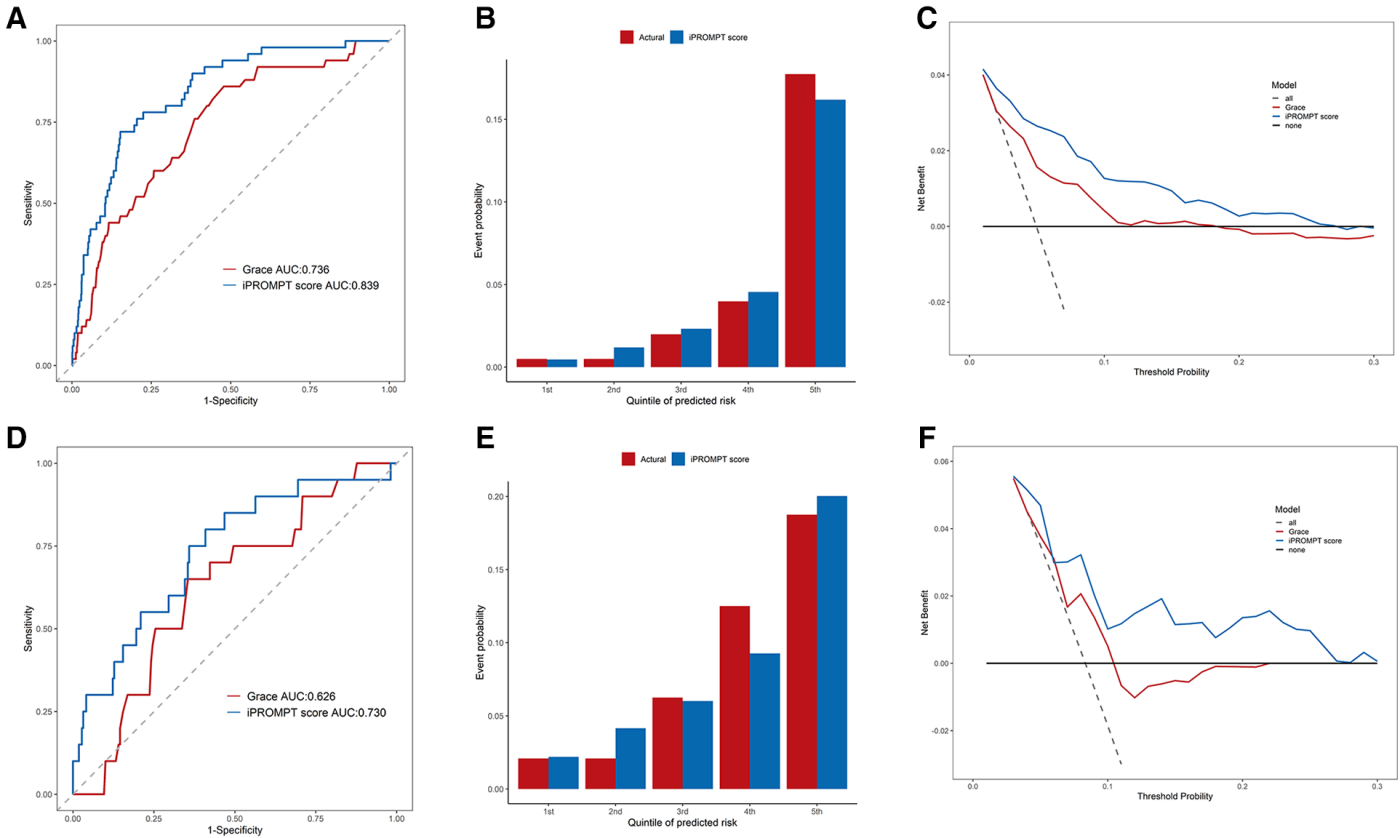
Performance evaluation of iPROMPT score and GRACE score in derivation cohort and external validation cohort. (**A,D**) Receiver-operating characteristic curve analysis. The accuracy for MACEs between the iPROMPT score and the GRACE risk score in the in derivation cohort (**A**) and external validation cohort (**D**). (**B,E**) The calibration plot shows the relationship between the observed and predicted proportion of events, grouped by quintile of risk in the in derivation cohort (**B**) and external validation cohort (**E**). (**C,F**) DCA curves for validating the clinical utility of the iPROMPT score and the previous model. AUC, area under the curve; GRACE, the Global Registry of Acute Coronary Events risk score; MACEs, major adverse cardiovascular events; DCA, decision curve analysis.

The iPROMPT score demonstrated good predictive value, with an improvement in the AUC compared with the GRACE risk score {from 0.736 [95% confidence interval (CI): 0.667–0.805] to 0.839 [95% CI: 0.786–0.892]} in the entire derivation cohort (*P* = 0.001, Figure 2A). The iPROMPT score also demonstrated better reclassification [NRI = 0.872 (95% CI: 0.616–1.127), *P* < 0.001; IDI = 0.067 (95% CI: 0.036–0.097), *P* < 0.001; Supplementary Table S4]. Calibration plots showed acceptable agreement between the iPROMPT score prediction and the actual observation in the derivation cohort (Figure 2B). Furthermore, we established a decision analysis curve to assess the net benefit to the decision, observing a higher net benefit of the iPROMPT score at all threshold probabilities (Figure 2C).

### 3.4. Variable importance and their association with MACEs

**Figure 3:**
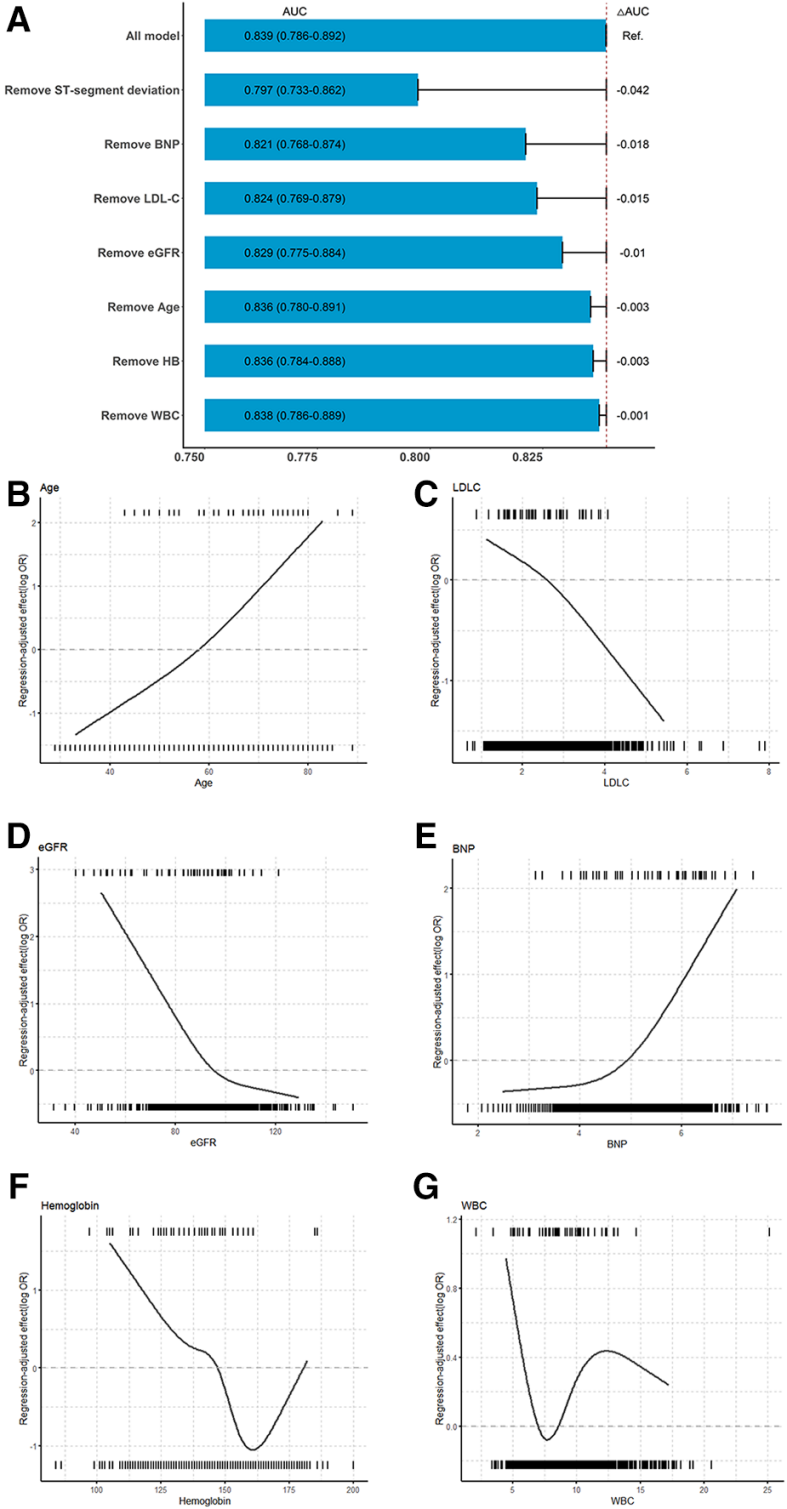
Association between continuous predictors and MACEs and importance of selected variables in iPROMPT. (**A**) The contribution of each selected variables in the iPROMPT score. (**B–G**) Regression-adjusted effects of selected continuous covariates in iPROMPT showed their association with MACEs following STEMI. The top of each figure shows the observed values of the continuous risk predictor among STEMI patients in the derivation cohort who experienced MACEs and the bottom shows the observed values of the predictor among those who did not experience MACEs. MACEs risk increased with age (**B**) and decreased LDL-C (**C**). MACEs risk declined with eGFR (**D**), and plateaued at over 100 ml/min/1.73 m^2^. (**E**) The risk associated with log(BNP) remained flat and increased when log(BNP) >5. The U-shaped association observed between hemoglobin (**F**), and WBC (**G**) and MACEs. AUC, area under the curve; BNP, B-type natriuretic peptide; eGFR, estimate glomerular filtration rate; WBC, white blood cell count.

To further ascertain how each variable contributed to the model, we assessed the variable importance according to the decrement in the AUC when the variable was replaced (Figure 3A). Seven variables were included in the iPROMPT score, including ST-segment deviation, BNP concentration, low-density lipoprotein cholesterol (LDL-C) concentration, eGFR, age, hemoglobin concentration, and white blood cell (WBC) count, which were ranked in descending order of importance.

The regression-adjusted association of continuous risk factors with MACEs following STEMI helped to interpret the complex ML models (Figures 3B–G). The risk increased with age and decreased with LDL-C concentration. However, the negative correlation observed between the eGFR and MACEs risk plateaued at an eGFR of >100 ml/min/1.73 m^2^. In contrast, the risk remained stable at log-transformed BNP concentration less than or equal to 5, and was inversely associated with MACEs risk as the slope increased. The association between the hemoglobin concentration and MACEs risk was U-shaped. Moreover, with an increase in the WBC count, MACEs risk increased initially and gradually decreased until leveling out at a WBC count of >12 × 10^9^/L.

### 3.5. Performance and variable contribution in subgroups

**Figure 4:**
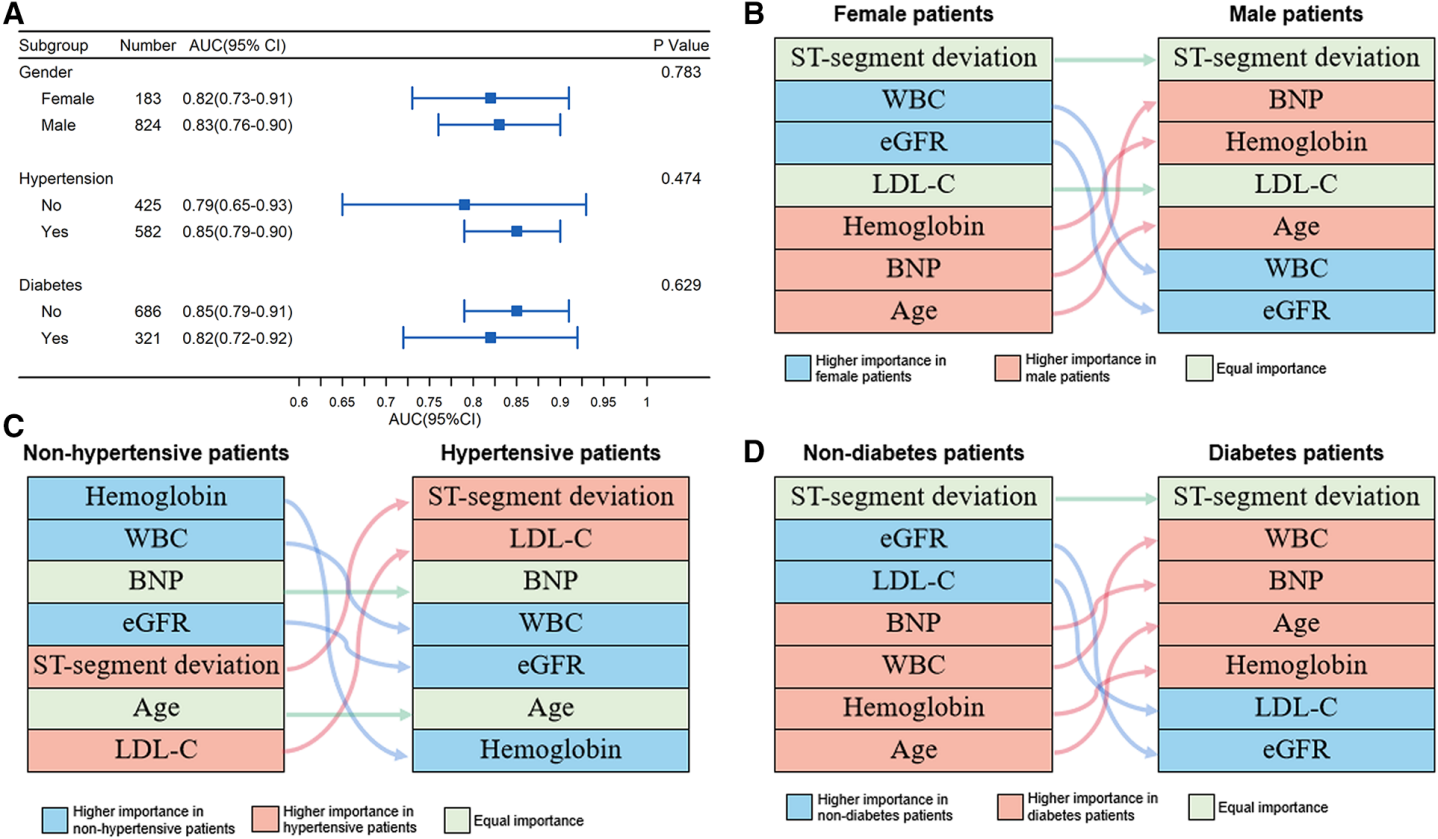
Performance and relative importance of variables in subgroups. (**A**) Forest plot for AUC and 95% confidence interval depicting overall discriminative efficacy of the iPROMPT score across pre-determined subgroups; (**B–D**) Alluvial plot of predictors for prognosis prediction identified by variables’ contribution in sex (**B**), hypertensive (**C**) and diabetic subgroup (**D**). AUC, area under the curve; BNP, B-type natriuretic peptide; eGFR, estimate glomerular filtration rate; WBC, white blood cell count.

Relevant risk factors, including sex, hypertension, and diabetes mellitus, were not included in the model. Thus, we conducted subgroup analyses based on these covariates. The performance of the iPROMPT score across predetermined subgroups was comparable (Figure 4A).

In each subgroup, the relative importance ranking of the variables was displayed in an alluvial plot (Figures 4B–D). The ST-segment deviation was the most important variable for risk prediction in both sexes, followed by the WBC count and eGFR in female patients, and BNP concentration and hemoglobin concentration in male patients. Hemoglobin concentration, WBC count, and eGFR showed a greater contribution in non-hypertensive patients, while ST-segment deviation and LDL-C concentration were more important predictors in patients with hypertension. Similar to sex stratification, ST-segment deviation was the top contributor in the subgroup stratified by diabetes mellitus. Compared with patients without diabetes mellitus, the WBC count, BNP concentration, age, and hemoglobin concentration were better predictors in patients with diabetes mellitus, but this was not the case for eGFR and LDL-C concentration. All aforementioned subgroup analysis results are shown in Supplementary Table S5.

### 3.6. Performance of the iPROMPT score in the external validation cohort

For external validation, we analyzed 240 patients with STEMI who attended the First Hospital of Jilin University. Of these patients, 20 (8.33%) experienced MACEs over the 2.84-year follow-up period. Several differences existed in clinical characteristics between the external validation and derivation cohorts (Supplementary Table S6).

Patients in the external validation cohort were assessed using the iPROMPT score. As expected, the iPROMPT score demonstrated improved model performance than the GRACE risk score [increase in the AUC to 0.730 (95% CI: 0.611–0.849), *P* = 0.010; NRI = 0.727 (95% CI: 0.293–1.162), *P* = 0.001; IDI = 0.062 (95% CI: 0.020–0.103), *P* = 0.004; Figure 2D]. When stratified by quintiles of event probability, similar predicted and observed frequencies indicated good score calibration (Figure 2E). Furthermore, the DCA showed higher clinical utility of the iPROMPT score in the external validation cohort (Figure 2F).

### 3.7. Development of the online dynamic nomogram

For physicians and patients alike, a web-based tool would be attractive to avoid tedious and time-consuming calculation. Thus, we built a web-based calculator (https://rebaccacwy.shinyapps.io/iPROMPTscore/) to predict MACEs risk in individual patients with STEMI (Supplementary Figure S4).

## 4. Discussion

In this study, seven practical variables were identified from routine clinical data to develop an ML-based risk prediction model (the iPROMPT score). The iPROMPT score demonstrated improved performance than the GRACE risk score in predicting MACEs following STEMI in the entire cohort and in subgroups. Moreover, distinct contributions of the selected variables among sex, hypertension, and diabetes mellitus subgroups were observed. To improve the clinical utility, we constructed a personalized and user-friendly web-based nomogram to help physicians with earlier individualized management in patients with STEMI.

### 4.1. Selected variables mirror critical pathophysiological processes following STEMI

The iPROMPT score uses seven clinical variables, including ST-segment deviation, BNP concentration, LDL-C concentration, eGFR, age, hemoglobin concentration, and WBC count, which are routinely assessed and readily available from electronic medical records. The chosen variables included in the iPROMPT score may mirror underlying pathological processes following STEMI, including cardiac injury, aging, renal damage, metabolic disorder, neuroendocrine deregulation, and inflammation, amongst others.

**ST-segment deviations** caused by loss of the cell membrane in the injured myocardium (18) had the greatest contribution in our model. Of note, ST-segment deviations are essential in standardized guidelines for the diagnosis of myocardial ischemia, the assessment of ischemia severity, and the prognosis evaluation (19). Consistent with a previous report (20), we demonstrated that **age** is a risk factor for MACEs prediction in patients with STEMI. Previous studies have demonstrated that renal dysfunction is an important risk factor after acute MI (21). We selected **eGFR** as a key index of renal function, which was corrected for age and sex, and more objectively replaced the creatinine concentration evaluated in the GRACE score.

The predictive value of the **hemoglobin** concentration in MI prognosis has been reported (22, 23). Here, a similar U-shaped association between the hemoglobin concentration at admission and MACEs following STEMI was observed (24). A reduction in the hemoglobin concentration lowers the oxygen-carrying capacity of the blood, which is compensated for by an increase in cardiac output (25). Such compensatory mechanisms are partly regulated by sympathetic activation (26), which is detrimental in patients with coronary artery disease. In addition, as cardiac output increases, mortality increases following left ventricular systolic dysfunction. Moreover, hemoglobin scavenges endogenous nitric oxide, leading to vasoconstriction and cardiovascular complications (27). In addition, excess cell-free hemoglobin is toxic because of its pro-oxidant and pro-inflammatory activity (28).

**BNP** and its counterpart NT-pro-BNP are neurohormones that are synthesized predominantly in the ventricular myocardium, and increases in their concentration reveal cardiac neurohormonal activation after myocardial damage (26). As an important indicator of cardiac function, a higher BNP concentration reflects impaired ventricular function and indicates the degree of myocardial injury (29). Consistent with previous reports, a higher BNP concentration upon admission is a powerful prognostic marker associated with an increased risk of MACEs following STEMI.

A U-shaped relationship was observed between **the WBC count** and long-term MACEs after STEMI. Leukocytes carry information about systemic inflammation, and MI triggers an intense inflammatory response that is essential for cardiac repair. However, exaggerated inflammation is detrimental to cardiac repair. Excessive inflammation indicated by increased leukocytes is harmful in patients with STEMI. Several hypotheses underlying such associations have been postulated such as leukocyte-mediated microcirculatory malperfusion (30), excessive thrombus formation (31), and indirect cardiotoxicity through pro-inflammatory cytokines (32).

Elevated **LDL-C** is a well-established risk factor for cardiovascular disease. However, despite having a higher LDL-C at admission, patients paradoxically had better outcomes, which is supported by previous reports (33). Such paradoxes may be explained by the following factors. First, lipid concentrations are significantly influenced by body mass index, presenting a similar “obesity paradox” (34, 35), which is largely explained by reverse causation. Second, low LDL-C reflects a poor nutritional status, which is associated with a decline in functional performance or increased frailty (36). Third, LDL-C is critical for cell membrane synthesis, and an extremely low LDL-C concentration is harmful to cell survival (37). Meanwhile, the greater sympathetic activity triggered by acute MI directly provokes hydrolyzation of lipoprotein triglycerides by activated lipoprotein lipase (38). In addition, inflammation-induced lipid reduction underlies the lipid paradox postulated in previous studies (39).

### 4.2. The iPROMPT score provides complementary information to pre-existing models

The ML algorithms provided additional pathophysiologic information to the standard regression analysis alone. The variables included in the iPROMPT score overlapped with the predictors in previous models. Additional parameters in the iPROMPT score that describe pathways, such as WBC count (inflammatory response), hemoglobin concentration, and BNP concentration (neurohormonal activation), provide complementary information to the GRACE score. Recently D'Ascenzo and colleagues proposed an ML-based predictive model for acute coronary syndrome (ACS) (the PRAISE score) that stratifies patients according to ischemic and bleeding risk (40). Hemoglobin concentration, age, and eGFR emerged as overlapping features in both the iPROMPT and PRAISE scores and demonstrated critical processes for recurrent ischemic events. Nevertheless, the PRAISE score was developed based on a heterogeneous population of patients with ACS, in whom complete revascularization as a covariate increased the risk of ischemic events. We focused on the prediction in patients with STEMI undergoing PCI to understand the residual risk after revascularization. Several investigations have focused on risk stratification in patients with STEMI using ML algorithms (41, 42). Nevertheless, only a few studies have focused on the long-term risk following MACEs (43, 44). An ML model to predict 1-year mortality after acute STEMI was developed and outperformed the existing risk score, with an AUC of 0.84 (95% CI: 0.798–0.872) (43). The model included 12 clinical features that did not represent a valid simplification in clinical decision-making Conversely, the iPROMPT score can be computed with only seven variables that are largely available in routine clinical practice and that yield comparable performance.

### 4.3. Performance differences in the iPROMPT score among subgroups

Given that STEMI patients who are stratified into subgroups according to traditional risk factors, including sex, hypertension, and diabetes mellitus, have distinct clinical profiles, resulting in different MACEs risks. Whereas those risk factors, including sex, hypertension, and diabetes mellitus, were not included in the model. We further evaluated the performance of the iPROMPT score in subgroups defined by confirmed risk factors, including sex, hypertension, and diabetes mellitus. We identified distinct contributions of the selected variables across subgroups, which implicated different pathophysiological processes in prespecified subgroups.

According to previous studies, the prognosis of women with MI is worse than that of men (45, 46). However, the underlying mechanisms explaining the increase in the cardiovascular vulnerability of women are not yet understood. The adverse effects of estrogen withdrawal on cardiovascular health probably act *via* alterations in body fat distribution, endothelial dysfunction, vascular inflammation, and sympathetic tone (45). Such processes may correlate with the eGFR and/or WBC count, which were of higher importance in female patients in our subgroup analysis. In addition, emotional stress-induced amygdalar activity leading to upregulated inflammatory states negatively affects myocardial function and perfusion in a sex-dependent manner (47). Moreover, a detrimental association between inflammation and adipose tissue has been speculated to underlie worse outcomes in women (48, 49).

Antecedent hypertension, which is a well-established cardiovascular risk factor, is associated with adverse outcomes in acute MI survivors. The clustering of traditional cardiovascular risk factors, including hypercholesterolemia, partly explains the higher risk of hard endpoints in hypertensive patients with STEMI (10). LDL-C concentration revealed cardiometabolic disturbances and showed great importance in hypertensive patients with STEMI in the present study. A higher diastolic pressure leads to abundant collateral circulation with a subsequent improvement in reperfusion efficacy and limited myocardial salvage (49). Moreover, microvascular damage within culprit arteries is a mechanism underpinning the poor prognosis of hypertensive patients after acute MI (50). The resolution of ST-segment deviation as a marker of microvascular recovery (51) is associated with better outcomes after STEMI. Therefore, it is no surprise that ST-segment deviation had great importance in the hypertensive subgroup.

Compared with patients without diabetes mellitus, the WBC count, BNP concentration, age, and hemoglobin concentration suggested that inflammatory, neuroendocrine, and aging pathways are essential in patients with diabetes mellitus after acute STEMI onset. Increasing evidence suggests that diabetes mellitus is associated with vascular inflammation (52). A positive correlation between fasting glucose and BNP concentrations has been reported previously (53). As speculated, a higher plasma glucose concentration may induce a hypertonic state, increase ventricular tension, increase BNP concentration, and eventually accelerate neurohormonal changes after acute STEMI (53). Aging is a primary risk factor for diabetes mellitus (54), and accelerated aging may worsen the prognosis of patients with STEMI. The role of hemoglobin has been discussed above; however, chronic hyperglycemia in patients with diabetes mellitus can result in abnormal erythrocytes, oxidative stress, and sympathetic denervation of the kidney related to autonomic neuropathy (55), resulting in impaired erythropoietin production and changes in hemoglobin.

## 5. Strengths

Our findings highlight that the iPROMPT score, which uses fewer routinely assessed clinical variables, provides individual MACEs risk prediction in patients following STEMI, and has incremental prognostic value to the GRACE score. Although comparable performance between subgroups was observed, the variable importance ranking highlights the distinct contributions of cardiac injury (ST-segment deviation), age, hemoglobin concentration, renal damage (eGFR), metabolic disorder (LDL-C concentration), neuroendocrine deregulation (BNP concentration), and inflammation (WBC count) in different subgroups. Such data are valuable for planning of specific patient follow-ups and counseling. Furthermore, clinical variables included in the iPROMPT score are common in clinical practice and can be obtained rapidly after admission at both primary and tertiary hospitals. We constructed a web-based dynamic nomogram for the iPROMPT score, which avoids tedious calculations and potentially encourages the use of the model in clinical practice. Therefore, the clinician could pay close attention to patients with high MACEs risk evaluated by iPROMPT score and provide more frequent follow-up visits to improve their outcomes.

## 6. Limitations

Despite the significance of the findings, this study has some limitations that should be noted. First, although the patients included in this study were enrolled from two centers and the iPROMPT score was validated externally, further validation in a larger cohort is required for a higher level of confidence. Second, this study was retrospective; therefore, prospective studies should be performed in the future. Finally, only variables that are readily available in clinical practice were used in this study, and some potentially relevant variables, such as novel multi-omics biomarkers, could further improve the predictive power of the model.

## 7. Conclusion

In conclusion, we developed the iPROMPT score using readily available clinical variables to predict MACEs in patients following STEMI. The iPROMPT score demonstrated incremental prognostic value over the established GRACE risk score. We also observed comparable model performance and distinct contributions of selected variables in predetermined subgroups, which aids the understanding of the pathophysiological processes involved in different subgroups.

## Data Availability

The original contributions presented in the study are included in the article/**Supplementary Material**, further inquiries can be directed to the corresponding author/s.
