# Supplementary material for: Prediction models for major adverse cardiovascular events following ST-segment elevation myocardial infarction and subgroup-specific performance
Source: Front Cardiovasc Med. 2023 Apr 25;10:1181424. doi: 10.3389/fcvm.2023.1181424 (PMC10167292; doi:10.3389/fcvm.2023.1181424)
Supplement: Supplementary file 1 [file Table1.docx]

Supplementary Material

# Supplementary Figures and Tables

## Supplementary Figure1

**Supplementary Figure1. Flow chart for inclusion of STEMI patients from Beijing Anzhen Hosipital.**

## Supplementary Figure2

**
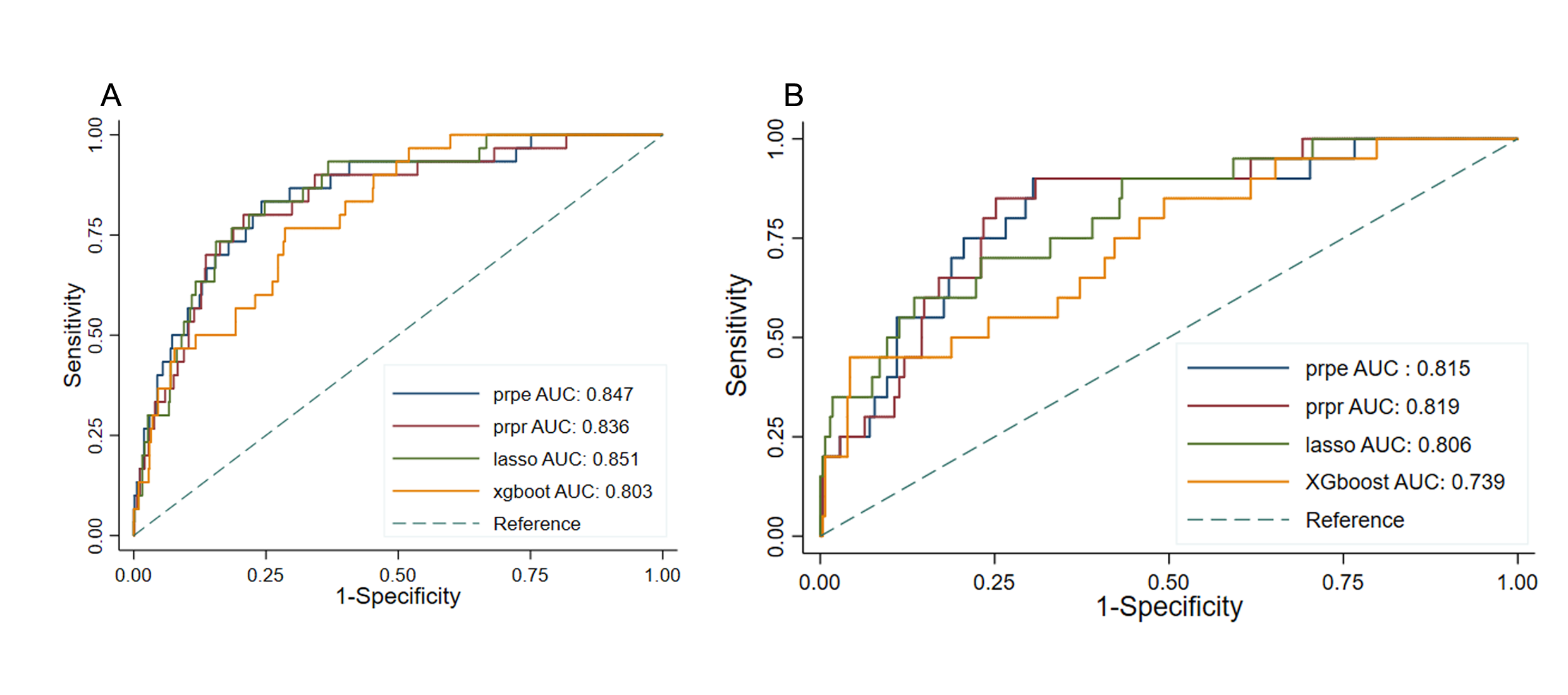
**

**Supplementary Figure2. Performance of different machine learning models in the training and internal validation datasets.** Comparing the area under the receiver operating characteristic curve of each model in the training(A) and internal validation datasets(B). prpe, forward stepwise model; prpr, backward stepwise model; lasso, Lasso regression model; Xg, XG Boost model; AUC, area under the curve.

## Supplementary Figure3

**
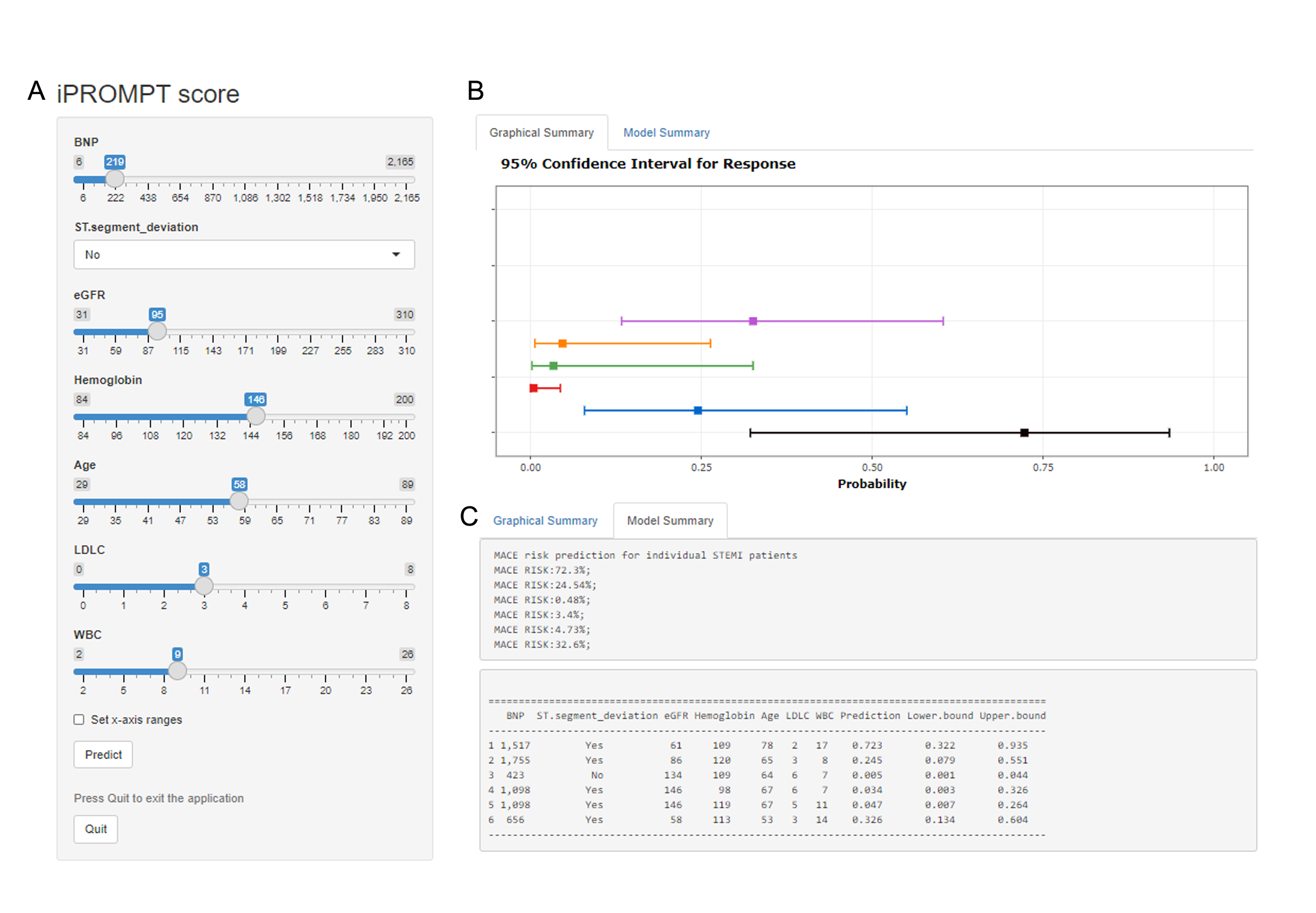
**

**Supplementary Figure3 .Web-based dynamic nomogram for MACEs risk prediction following acute STEMI.** Web-based dynamic nomogram for predicting major cardiovascular events in patients following acute STEMI. By entering the individual information of a STEMI patient, we could obtain corresponding risk probability. A Entering Interface: Individuals enter their personal details in this interface. B Graphical Summary: The probability of MACEs and 95% confidence interval of participants are depicted in this interface.C Numerical Summary: The actual values of probability developing MACEs and 95% confidence interval are shown in this interface. BNP, B-type natriuretic peptide; eGFR,estimate glomerular filtration rate;WBC, white blood cell count.

## Supplementary Table1

**Supplementary Table1 Missing value for the training dataset.**

| **Variable** | **Missing** | **Total** | **Percent Missing** |
| --- | --- | --- | --- |
| **BMI** | 113 | 705 | 16.03 |
| **CK-MB** | 4 | 705 | 0.57 |
| **ALT** | 3 | 705 | 0.43 |
| **AST** | 3 | 705 | 0.43 |
| **Mg** | 83 | 705 | 11.77 |
| **P** | 83 | 705 | 11.77 |
| **Ca** | 83 | 705 | 11.77 |
| **WBC** | 2 | 705 | 0.28 |
| **RBC** | 2 | 705 | 0.28 |
| **PLT** | 2 | 705 | 0.28 |
| **HB** | 2 | 705 | 0.28 |
| **NLR** | 2 | 705 | 0.28 |
| **PLR** | 2 | 705 | 0.28 |
| **hsCRP** | 60 | 705 | 8.51 |
| **TG** | 23 | 705 | 3.26 |
| **TC** | 23 | 705 | 3.26 |
| **HDL-C** | 23 | 705 | 3.26 |
| **LDL-C** | 23 | 705 | 3.26 |
| **non HDL-C** | 23 | 705 | 3.26 |
| **HCY** | 77 | 705 | 10.92 |
| **PT** | 23 | 705 | 3.26 |
| **PT%** | 23 | 705 | 3.26 |
| **INR** | 23 | 705 | 3.26 |
| **APTT** | 23 | 705 | 3.26 |
| **FBG** | 23 | 705 | 3.26 |
| **D-Dimer** | 10 | 705 | 1.42 |
| **FDP** | 24 | 705 | 3.4 |
| **E'wave** | 71 | 705 | 10.07 |
| **A'wave** | 85 | 705 | 12.06 |
| **E/A** | 85 | 705 | 12.06 |

BMI, body mass index; CK-MB, creatine kinase MB; ALT, alanine aminotransferase; AST, aspartate aminotransferase; Mg, magnesium; P, phosphorus; Ca, calcium; WBC, white blood cell count; RBC, red blood cell count; PLT, platelet; HB, hemoglobin; NLR, neutrophil-to-lymphocyte ratio; PLR, platelet-to-lymphocyte ratio; TG, triglycerides; TC, total cholesterol; LDL-C, low-density lipoprotein cholesterol; HCY, homocysteine; PT, prothrombin time; INR, international normalized ratio; APTT, activated partial thromboplastin time; FBG, fibrinogen; FDP, fibrin/fibrinogen degradation products; E/A, ratio of E'wave and A'wave.

**Supplementary Table2 Missing value for the internal validation dataset.**

| **Variable** | **Missing** | **Total** | **Percent Missing** |
| --- | --- | --- | --- |
| **BMI** | 57 | 302 | 18.87 |
| **CK-MB** | 1 | 302 | 0.33 |
| **BNP** | 36 | 302 | 11.92 |
| **Mg** | 35 | 302 | 11.59 |
| **P** | 35 | 302 | 11.59 |
| **Ca** | 35 | 302 | 11.59 |
| **TG** | 8 | 302 | 2.65 |
| **TC** | 8 | 302 | 2.65 |
| **HDL-C** | 8 | 302 | 2.65 |
| **LDL-C** | 8 | 302 | 2.65 |
| **non HDL-C** | 8 | 302 | 2.65 |
| **hsCRP** | 26 | 302 | 8.61 |
| **HCY** | 34 | 302 | 11.26 |
| **HbA1C** | 48 | 302 | 15.89 |
| **PT** | 10 | 302 | 3.31 |
| **PT%** | 10 | 302 | 3.31 |
| **INR** | 10 | 302 | 3.31 |
| **APTT** | 10 | 302 | 3.31 |
| **FBG** | 10 | 302 | 3.31 |
| **D-Dimer** | 4 | 302 | 1.32 |
| **FDP** | 10 | 302 | 3.31 |
| **LVESD** | 18 | 302 | 5.96 |
| **E'wave** | 30 | 302 | 9.93 |
| **A'wave** | 40 | 302 | 13.25 |
| **E/A** | 40 | 302 | 13.25 |

BMI, body mass index; CK-MB, creatine kinase MB; BNP, B-type natriuretic peptide; Mg, magnesium; P, phosphorus; Ca, calcium; TG, triglycerides; TC, total cholesterol; LDL-C, low-density lipoprotein cholesterol; hsCRP, hypersensitive C-reactive protein; HCY, homocysteine; HbA1c, glycated hemoglobin A1c; PT, prothrombin time; INR, international normalized ratio; APTT, activated partial thromboplastin time; FBG, fibrinogen; FDP, fibrin/fibrinogen degradation products; LVESD, left ventricular end systolic diameter; E/A, ratio of E'wave and A'wave.

**Supplementary Table3 Performance metrics of different machine learning models.**

|  | **GRACE score** | **Stepwise Forward** |  | **Stepwise Backward** |  | **LASSO** |  | **XGBoost** |  |
| --- | --- | --- | --- | --- | --- | --- | --- | --- | --- |
| **AUC** |  |  |  |  |  |  |  |  |  |
| **Training dataset** | 0.748  (0.665-0.830) | 0.847  (0.776-0.918) | 0.022 | 0.836  (0.763-0.910) | 0.046 | 0.851  (0.787-0.914) | 0.017 | 0.803  (0.735-0.872) | 0.243 |
| **Testing dataset** | 0.715  (0.588-0.841) | 0.818  (0.721-0.914) | 0.025 | 0.819  (0.732-0.906) | 0.010 | 0.805  (0.705-0.905) | 0.034 | 0.747  (0.632-0.862) | 0.623 |
| **NRI** |  |  |  |  |  |  |  |  |  |
| **Training dataset** | reference | 0.699  (0.334-1.065) | 0.000 | 0.562  (0.196-0.927) | 0.003 | 0.604  (0.239-0.970) | 0.001 | 0.633  (0.267-0.998) | 0.001 |
| **Testing dataset** | reference | 0.334  (-0.119-0.788) | 0.149 | 0.327  (-0.127-0.780) | 0.158 | 0.726  (0.273-1.180) | 0.002 | 0.554  (0.100-1.007) | 0.017 |
| **IDI** |  |  |  |  |  |  |  |  |  |
| **Training dataset** | reference | 0.093  (0.020-0.166) | 0.013 | 0.063  (0.008-0.119) | 0.025 | 0.062  (0.001-0.122) | 0.047 | 0.068  (-0.003-0.139) | 0.060 |
| **Testing dataset** | reference | 0.076  (0.012-0.140) | 0.020 | 0.058  (0.009-0.107) | 0.021 | 0.146  (0.048-0.243) | 0.004 | 0.071  (0.018-0.123) | 0.009 |

AUC, area under the curve; NRI, net reclassification improvement; IDI, integrated discrimination improvement.

**Supplementary Table4 Improved model performance over the GRACE score.**

|  | **GRACE score** | **iPROMPT score** | **P value** |
| --- | --- | --- | --- |
| **AUC** | | | |
| **Deviation cohort** | 0.736(0.667-0.805) | 0.839(0.786-0.892) | 0.002 |
| **External validation cohort** | 0.626(0.508-0.743) | 0.730(0.611-0.849) | 0.010 |
| **NRI** | | |  |
| **Deviation cohort** | reference | 0.872(0.616-1.127) | <0.001 |
| **External validation cohort** | reference | 0.727(0.293-1.162) | 0.001 |
| **IDI** | | |  |
| **Deviation cohort** | reference | 0.067(0.036-0.097) | <0.001 |
| **External validation cohort** | reference | 0.062(0.020-0.103) | 0.004 |

AUC, area under the curve; NRI, net reclassification improvement; IDI, integrated discrimination improvement.

**Supplementary Table5 Model performance and variables’ importance in subgroup analysis**

|  | **AUC** | **Importance (∆AUC)** | **AUC** | **Importance (∆AUC)** |
| --- | --- | --- | --- | --- |
|  | **Non-hypertensive patients** | | **Hypertensive patients** | |
| All model | 0.883(0.830-0.936) | Ref | 0.858(0.806-0.909) | Ref |
| Remove BNP | 0.868(0.795-0.941) | -0.014 | 0.844(0.792-0.897) | -0.013 |
| Remove ST-segment deviation | 0.876(0.813-0.939) | -0.007 | 0.795(0.720-0.870) | -0.063 |
| Remove eGFR | 0.875(0.821-0.929) | -0.008 | 0.851(0.800-0.903) | -0.006 |
| Remove Hemoglobin | 0.853(0.779-0.927) | -0.030 | 0.856(0.805-0.907) | -0.002 |
| Remove Age | 0.883(0.831-0.935) | 0.000 | 0.854(0.799-0.909) | -0.004 |
| Remove LDL-C | 0.884(0.831-0.936) | 0.001 | 0.832(0.773-0.890) | -0.026 |
| Remove WBC | 0.861(0.798-0.924) | -0.022 | 0.848(0.794-0.903) | -0.010 |
|  |  |  |  |  |
|  | **Non-diabetic patients** | | **Diabetic patients** | |
| All model | 0.860(0.803-0.917) | Ref | 0.851(0.756-0.946) | Ref |
| Remove BNP | 0.848(0.791-0.905) | -0.012 | 0.835(0.737-0.934) | -0.016 |
| Remove ST-segment deviation | 0.833(0.757-0.910) | -0.027 | 0.810(0.713-0.908) | -0.041 |
| Remove eGFR | 0.839(0.778-0.900) | -0.021 | 0.851(0.755-0.946) | 0.000 |
| Remove Hemoglobin | 0.859(0.808-0.911) | 0.000 | 0.844(0.752-0.936) | -0.007 |
| Remove Age | 0.862(0.805-0.919) | 0.002 | 0.843(0.744-0.942) | -0.008 |
| Remove LDL-C | 0.842(0.776-0.909) | -0.017 | 0.847(0.756-0.938) | -0.004 |
| Remove WBC | 0.857(0.797-0.918) | -0.003 | 0.829(0.727-0.931) | -0.022 |
|  |  |  |  |  |
|  | **Female patients** | | **Male patients** | |
| All model | 0.892(0.817-0.968) | Ref | 0.842(0.771-0.913) | Ref |
| Remove BNP | 0.883(0.801-0.966) | -0.009 | 0.827(0.755-0.899) | -0.015 |
| Remove ST-segment deviation | 0.830(0.736-0.925) | -0.062 | 0.824(0.745-0.902) | -0.019 |
| Remove eGFR | 0.857(0.754-0.960) | -0.035 | 0.840(0.768-0.912) | -0.002 |
| Remove Hemoglobin | 0.875(0.796-0.954) | -0.017 | 0.831(0.762-0.901) | -0.011 |
| Remove Age | 0.893(0.818-0.968) | 0.001 | 0.836(0.760-0.912) | -0.006 |
| Remove LDL-C | 0.875(0.796-0.953) | -0.018 | 0.835(0.767-0.904) | -0.007 |
| Remove WBC | 0.853(0.759-0.947) | -0.039 | 0.840(0.770-0.910) | -0.002 |

**Supplementary Table6 Baseline characteristics of included STEMI patients according to the** deviation **cohort and external validation cohort.**

|  | Deviation （N=1007） | **External** Validation （N=240） |
| --- | --- | --- |
| Demographic characteristic |  |  |
| Male gender, n (%) | 824 (81.8%) | 173 (72.1%) |
| Age, years | 57.7±11.0 | 60.9±10.5 |
| Admission status |  |  |
| SBP, mmHg | 122±17.7 | 131±23.7 |
| DBP, mmHg | 74.5±11.6 | 78.6±13.5 |
| Heart rate, bpm | 73.4±12.0 | 77.0±14.1 |
| ST-segment deviation, n (%) | 344 (34.2%) | 191 (79.6%) |
| GRACE score | 123±33.1 | 143±33.7 |
| Killip classification |  |  |
| 1 | 872 (86.6%) | 166 (69.2%) |
| 2 | 101 (10.0%) | 59 (24.6%) |
| 3 | 10 (0.99%) | 6 (2.50%) |
| 4 | 24 (2.38%) | 9 (3.75%) |
| Cardiac arrest, n (%) | 20 (1.99%) | 10 (4.02%) |
| Multivessel disease, n (%) | 750 (74.5%) | 172 (71.7%) |
| Personal history |  |  |
| Hypertension, n (%) | 582 (57.8%) | 104 (43.3%) |
| Diabetes mellitus , n (%) | 321 (31.9%) | 47 (19.6%) |
| Hyperlipidemia , n (%) | 886 (88.0%) | 53 (22.1%) |
| Prior myocardial infarction, n (%) | 64 (6.36%) | 2 (0.83%) |
| Prior CVD, n (%) | 117 (11.6%) | 76 (31.7%) |
| Smoking, n (%) | 527 (52.3%) | 119 (49.6%) |
| Laboratory tests |  |  |
| cTnI, ng/l | 0.78 (0.05-11.7) | 4.82 (0.68-30.1) |
| BNP, pg/ml | 2.12±0.46 | NA |
| NT-proBNP, pg/ml | NA | 445 (195-1300) |
| eGFR(CKD-EPI) | 94.9±15.6 | 91.2±17.1 |
| LDL-C, mmol/l | 2.70±0.93 | 2.93±0.87 |
| White blood cell count, n/dl | 9.03±2.97 | 10.6±3.70 |
| Hemoglobin, g/l | 146±15.8 | 143±19.3 |
| Cardiac uhrasonography |  |  |
| Ejection fraction, % | 55.1±8.55 | 52.9±6.71 |
| LVEDi, mm | 48.8±5.00 | 49.0±5.14 |

For continuous variables, non-normal variables were expressed as the median [interquartile range (IQR)], and normal variables were expressed as the mean [standard deviation (SD)]. Categorical variables are expressed in N (%). SBP, systolic blood pressure; DBP, diastolic blood pressure; GRACE, Global Registry of Acute Coronary Events; CVD, cardiovascular disease; cTnI, cardiac troponin I; BNP, B-type natriuretic peptide; eGFR, estimate glomerular filtration rate; LDL-C, low-density lipoprotein cholesterol; LVEDi, left ventricle end-diastolic volume index.
